# Associations between Unsociability and Peer Problems in Chinese Children and Adolescents: A Meta-Analysis

**DOI:** 10.3390/bs14070590

**Published:** 2024-07-11

**Authors:** Na Hu, Wen Zhang, Aersheng Haidabieke, Jiyueyi Wang, Nan Zhou, Xuechen Ding, Hong Zheng

**Affiliations:** 1 School of Psychology, Shanghai Normal University, Shanghai 200234, China; huna54@shnu.edu.cn (N.H.); 1000498022@smail.shnu.edu.cn (A.H.); 1000514554@shnu.edu.cn (J.W.); 2 Lab for Educational Big Data and Policymaking, Ministry of Education of the People’s Republic of China, Shanghai 200234, China; 3 CAS Key Laboratory of Behavioral Science, Institute of Psychology, Chinese Academy of Sciences, Beijing 100101, China; zhangwen@psych.ac.cn; 4 Department of Psychology, University of Chinese Academy of Sciences, Beijing 100049, China; 5 College of Preschool Education, Capital Normal University, Beijing 100048, China; nanzhou@cnu.edu.cn; 6 The Research Base of Online Education for Shanghai Middle and Primary Schools, Shanghai 200234, China; 7 Shanghai Changning Mental Health Center, Shanghai 200335, China

**Keywords:** unsociability, peer problems, Chinese culture, meta-analysis, children, adolescents

## Abstract

Research has shown that unsociability, reflected as a personal choice, is not necessarily associated with socio-emotional problems in Western countries. However, the associations between unsociability and peer problems are consistently evident in Chinese culture, yet the strength and direction in these associations are mixed. The present study aimed to examine whether unsociability is associated with peer problems and explored the potential moderators among the associations. A meta-analysis was conducted using publications that measured unsociability and peer problems. A total of 21 articles involving 43 effect sizes from 12,696 Chinese children and adolescents were included. The results revealed that (1) unsociability was positively associated with peer problems (*r* = 0.32, *p* < 0.001) among children and adolescents. (2) Informants (i.e., self-reports, peer nominations, teacher ratings, and parent ratings) and living areas (i.e., urban, suburban, and rural areas) significantly moderated the associations between unsociability and peer problems. Specifically, the associations were stronger for peer-nominated unsociability, self-reported peer problems, and samples in suburban areas. These findings shed light on unsociability linked to higher levels of peer problems among Chinese children and adolescents. Still, the influences are unique to peer problems and moderated by both data sources and environmental factors.

## 1. Introduction

Socially withdrawn children tend to remove themselves from opportunities for peer interactions and frequently perform solitary behaviors in social contexts [1]. Although social withdrawal was generally linked to children’s social, emotional, and academic maladjustment [2], subtypes with different social approach and social avoidance motivations appear to be differentially associated with individuals’ adjustment outcomes [3,4]. Unlike sociability (i.e., preference for being with others rather than being alone) [5], unsociability, a subtype of social withdrawal representing a natural preference for solitude, is characterized by low social approach (i.e., the desire to look for social interactions) and low-to-average social avoidance motivations (i.e., the desire to avoid social interactions) [6,7], which increases the risk of children’s socio-emotional malfunctioning in social situations [3,4]. However, researchers have also found that unsociability does not appear to be directly linked to psychological maladjustment in Western cultures [8,9].

Although there are common associations between social withdrawal and maladjustment, cultural values play an important role in determining the meanings and implications of different types of social withdrawal [10]. In Western culture, influenced by individualism, individuals are encouraged to pursue their own goals and values, focusing on personal growth and self-actualization. Unsociability may not be seen as negative because it represents a personal choice and does not conflict with individualistic values. Conversely, in Eastern cultures, which tend to be influenced by collectivism, more emphasis is placed on the individual’s sense of belonging to a group and the need to follow social norms to foster good peer relationships [11]. Therefore, the associations between unsociability and peer problems are suggested to be largely shaped by socio-cultural contexts [12,13]. The large-scale social changes in China in the last decades have led to the co-junction of characteristics from different values in the Chinese context, providing a unique view to understanding the role of culture on unsociable children’s peer problems [14]. In contrast to findings from Western societies [15], a series of empirical studies have manifested the associations between unsociability and peer problems in the Chinese context. For example, unsociable Chinese children experience increased peer problems [16,17]. However, there is a lack of systematic review to understand the impact of unsociability on peer problems in China. Accordingly, the present study aimed to conduct a meta-analysis of unsociability and peer problems in the Chinese context.

### 1.1. Conceptualizations of Unsociability

Unsociability refers to the social disinterest and non-fearful preference for solitary activities, being indicative of a motivation to be alone that reflects the positive appeal that solitude and solitary activities hold for individuals [15]. It underscores the important notion that unsociable individuals have an intrinsic motivation for spending time alone [18]. Specifically, unsociable children perform withdrawn behaviors due to their preference and enjoyment of solitary activities [15,19]. The conceptualization of unsociability still lacks lucidity in the extant literature [15]. A number of terms have been conducted overlapping with unsociability, including preference for solitude [20,21], affinity for solitude [22], social disinterest [23], solitropic orientation [24], and affinity for aloneness [25,26]. The above terms belong to various aspects of solitude [27] and all share a common underlying theme of a non-fearful preference for spending time alone [15]. Since the current study focused on the motivational aspects of solitude, the above terms were all considered conceptually equivalent and were used to discuss the associations between unsociability and peer problems.

### 1.2. Associations between Unsociability and Peer Problems in China

Peer groups play an important and unique role in children’s social, emotional, cognitive, and moral development [28]. Children involved in infrequent social interactions may “miss out” on those benefits from peers, possibly leading to difficulties in social adjustment [2,29]. There are several indices of peer problems reflecting different processes of negative peer relationships, including peer rejection and peer victimization [30,31]. Peer rejection is constructed as encompassing peer behaviors to thwart one’s overtures [31], whereas peer victimization can be defined as being the target of intended peers’ hurtful behaviors [30]. A line of work has found that peer problems in childhood and adolescence may have strong associations with internalizing problems, which may turn out to be the risk factors for their socio-emotional adjustment in emerging adulthood [17,32,33,34]. For example, Xiao and colleagues also found that unsociability was negatively associated with subsequent loneliness through the mediating effect of peer rejection [16].

The meaning and adaptive value of certain social behaviors may vary across cultural contexts [35]; unsociability is viewed as a relatively benign form of solitary behavior in Western societies [8,9,19,29,36,37,38]. Solitary behaviors may reflect a personal choice, self-assertiveness, and autonomous action, which are conformed to their self-oriented or individualistic cultural values [39]. In addition, unsociable children and adolescents show social disinterest and non-fearful preference for solitude [3]. Therefore, spending time alone may provide more opportunities to improve self-identification and self-construction, ref. [25] attenuate the development of peer difficulties, and help those children and adolescents to be adaptive [15]. Unlike the emphasis on expressing personal choice in a self-oriented culture, Chinese culture is a typical group-oriented society that encourages social interdependence and group harmony [40,41,42]. Chinese children are encouraged to participate in group activities and contribute to collective wellbeing and the disengaging behaviors from peer groups in unsociable children and adolescents may be considered as anti-group, selfish, and abnormal [13]. Indeed, children who are not interested in group interaction and prefer to spend time alone are often viewed as deviant, criticized by adults, and disliked by peers [40], which in turn may contribute to the development of adjustment difficulties [43].

It should be noted that empirical studies found inconsistent evidence of the associations between unsociability and peer problems across different cultural contexts. Several studies in Western societies indicated that unsociability was not necessarily associated with peer problems [8,9,19,29,36,37,38]. However, unsociability in childhood and adolescence was found to be significantly associated with peer problems in the Chinese context, both concurrently and longitudinally [43,44,45,46,47,48]. A cross-cultural study directly compared the implications of unsociability among Chinese and Canadian children and adolescents and revealed a stronger association between unsociability and peer problems in Chinese children, as compared to their Canadian counterparts [49]. 

## 2. The Present Study

Although previous studies found positive associations between unsociability and peer problems in Chinese culture [50,51,52], the strength and direction of these associations were mixed and no study has synthesized the previous findings to examine the associations in a Chinese sample. Ran and colleagues conducted a meta-analysis to assess the associations between three subtypes of social withdrawal and peer problems in Chinese and North American youth to detect the mixed results in Western countries and China [53]. Despite the significant and positive associations suggesting social withdrawal was a risk factor for social adjustment in both Chinese and North American youth, this meta-analysis neglected the motivational and cultural specificity of unsociability. Considering the low approach that motivation, unsociability, and social avoidance fall under the broader construct of preference for solitude, it ignores the intrinsic motivation of unsociability [7]. Therefore, the previous meta-analysis on the associations between social withdrawal and peer problems is not able to understand how unsociability is related to peer problems in Chinese culture further. Accordingly, the present study aimed to address this issue by performing a meta-analysis of studies involving unsociability and peer problems in Chinese children and adolescents. As aforementioned, considering the positive associations between unsociability and peer problems in Chinese children and adolescents, we hypothesize that unsociability would be significantly and positively associated with peer problems, that is, children with higher levels of unsociability would have more peer problems.

Moreover, whether there are moderating factors among the associations between unsociability and peer problems were also examined in the present study. Preference for solitude has become increasingly adaptive and provides a context for independence and behavioral autonomy from childhood to adolescence [15]. From early adolescence, children may become increasingly attentive to endorsing individualistic values, which diminishes their negative response towards people with a higher level of unsociability, leading to weaker associations between unsociability and peer problems. Thus, age groups may buffer the negative effect of unsociability on peer problems. In addition, the progress of urbanization in China in the past four decades has made the population shift from rural to urban areas. Urban areas have become more modern and global, which makes it complicated to understand how Chinese cultures are influenced by Western cultures (e.g., independence and assertiveness), whereas rural areas have retained the traditional cultures (i.e., collectivism) emphasizing interpersonal harmony [35]. Previous studies found that unsociability was more strongly associated with peer problems in the rural context [54], while little attention has been paid to the associations in suburban areas. Compared to urban and rural areas, children and adolescents face constant changes in living conditions and challenges of mixed cultural values, which may lead them to be more maladjusted [43,55]. It should be also noted that multiple informants were used to measure unsociability and peer problems, including self-reports, peer nominations, parent ratings, and teacher ratings. However, most of the previous studies rely on one informant solely, which may influence the strength of the associations between unsociability and peer problems. Therefore, these potential moderators (e.g., age group, living areas, and informants) should be examined in the present meta-analysis. It is expected that the associations between unsociability and peer problems would be stronger for children and people living in suburban areas, and when unsociability and peer problems were measured by themselves.

## 3. Method

### 3.1. Study Selection

To retrieve relevant research on the associations between unsociability and peer problems interested in the present study, a systematic search was performed at the initial stage of study selection in various electronic databases: EBSCO, Sage, Science Direct, Springer Online Journals, Willey, China National Knowledge Infrastructure (CNKI), China Master’s Theses Full-text Database (CMFD), and the China Doctoral Dissertations Full-text Database (CDFD). Then, we manually checked the reference lists of selected studies and reviewed articles to reduce the likelihood of missing relevant studies.

To identify publications according to the following criteria, a screening process was undertaken, and identified papers were initially examined by two authors. The third independent author made a final decision. Considering that unsociability and solitude share a common construct of a non-fearful preference for spending time alone, we searched the published articles on the electronic databases mentioned above with the following combination of keywords in Chinese and English: (“social withdrawal” OR “unsociability” OR “solitude” OR “preference for solitude”) AND (“peer problems” OR “peer rejection” OR “peer victimization”).

**Figure 1:**
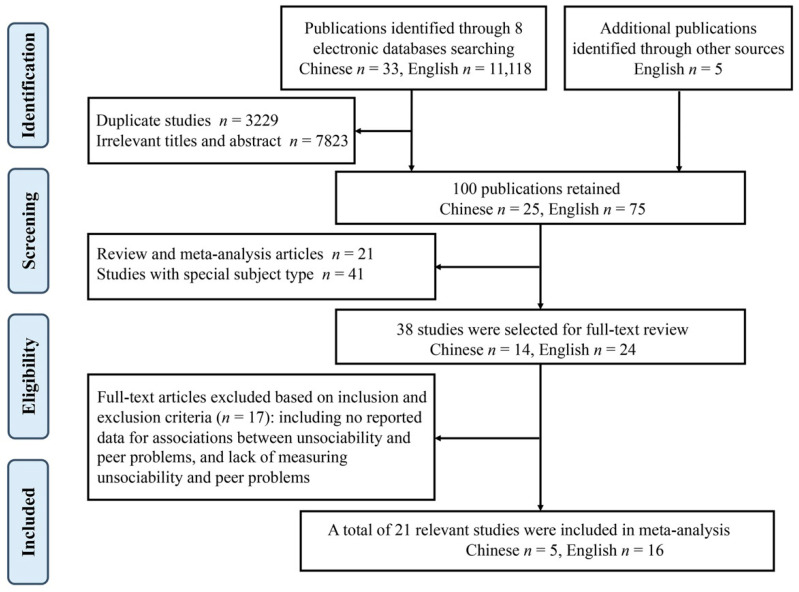
Flow diagram of the search procedure based on the sequence of exclusion and inclusion of eligible studies identified through the principal database searching.

All studies were included in the present meta-analysis if fulfilling the following criteria: (a) the study design was quantitative and empirical; review articles, qualitative studies, and case studies were excluded; (b) the studies investigated the associations between unsociability and peer problems; (c) the studies were written in English and Chinese (i.e., team members are fluent in both languages); (d) the studies included healthy participants without psychiatric diagnoses who were identified as school-aged children and adolescents with a mean age between 0 and 18 years from China; and (e) the studies reported statistical information to obtain or calculate at least one effect size (e.g., Pearson’s *r*). Figure 1 shows a flow chart of the search procedure. A total of 21 relevant publications were included in the final meta-analysis.

### 3.2. Coding of Variables

To synthesize the results from primary studies, each study was coded and extracted based on the following characteristics, including 43 effect sizes (see Table 1): (a) first author and year of publication; (b) number of participants (i.e., *N* = 12,696); (c) study design (i.e., cross-sectional or longitudinal); (d) gender (i.e., percentage of boys); (e) age group (i.e., children who are in kindergarten and elementary school or adolescents who are in middle and secondary school); (f) areas where participants live (i.e., urban, suburban, or rural regions with different industrialization and population size); (g) informants of unsociability (i.e., self-reports, peer nominations, or parent ratings); (h) informants of peer problems (i.e., self-reports, peer nominations, or teacher ratings); and (i) effect size (i.e., Pearson’s *r*). If the studies have more than one sample, effect sizes for each sample were included in the present meta-analysis. If the studies reported effect sizes for different subgroups, only effect sizes reported for the subgroups were included to reduce the likelihood of redundancy. If the studies reported multiple outcomes (such as peer rejection and peer victimization) or assessed unsociability and peer problems by multiple measures, all eligible effect sizes were included and coded. If different studies were reported on duplicate samples, only one study was included and coded. Two authors coded half of the selected studies separately and independently, then the third author reviewed the discrepancies, and the errors were corrected by consensus.

### 3.3. Statistical Analysis

The correlation coefficient (*r*) was chosen as the index of effect size in the present meta-analysis. In some cases, the studies did not report the correlation coefficients, and the Comprehensive Meta-Analysis software would compute Pearson’s r using the available statistical data reported in each study. We first estimated the overall associations between unsociability and peer problems. The positive r value indicates that a high level of unsociability is associated with a high level of peer problems. According to Cohen’s guidelines [56] (Cohen, 1977), the magnitude of the correlation was interpreted as small (0.10), medium (0.30), or large (0.50). Then, we conducted bivariate moderation analyses by using random effects models to examine the potential moderators on the associations between unsociability and peer problems.

Publication bias is a common concern for meta-analytic research [57], which means that studies reporting significant findings are more likely to be published than those reporting non-significant findings. In order to correctly evaluate the publication bias, we visually inspected the funnel plots of effect sizes and conducted Egger’s regression tests and the Begg and Mazumdar rank correlation test [58,59]. The significant *p*-value in both tests indicates that the publication bias may be detected. Additionally, Fail-Safe *N* was calculated to find the number of unpublished studies needed to change the effect size to non-significant [60]. All meta-analytic calculations were conducted using Version 3.0 of Comprehensive Meta-Analysis software [61].

## 4. Results

### 4.1. Heterogeneity Analyses and Publication Bias

A total of 21 published articles met the inclusion criteria, including 43 effect sizes (i.e., total *k* = 43) among 12,696 children and adolescents (i.e., total *N* = 12,696). The authors, publication years, sample sizes, and effect sizes of these studies are presented in Table 1, along with other important study characteristics (i.e., percentage of boys, living areas, age group, study design, and informants). According to Higgins and Thompson, the values of I2 around 25%, 50%, and 75% can be interpreted as low, medium, and high heterogeneity, respectively [62]. Thus, a heterogeneity analysis indicated a significant and high degree of heterogeneity with 96.98% of the observed variability attributable to systematic between-study differences (*I*^2^ = 96.98%), and the hypothesis that the effect was estimated as the same as the underlying population value could be rejected (*Q* (*df* = 42) = 1391.52, *p* < 0.001), which further suggested that our dataset should be analyzed through random effects models.

**Figure 2:**
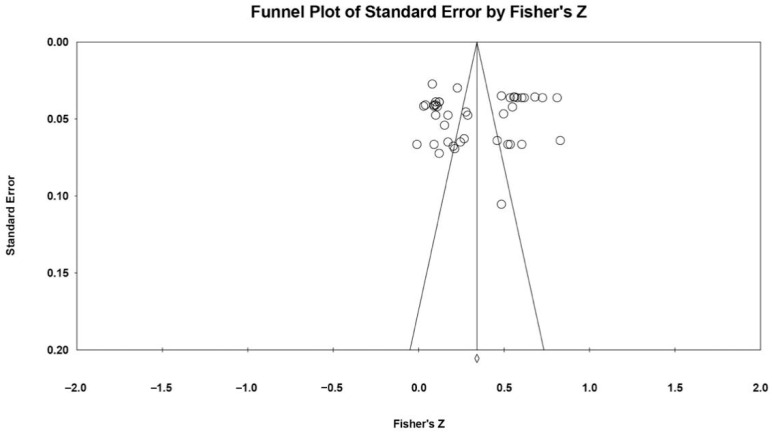
Funnel plot of effect sizes of the correlation between unsociability and peer problems in Chinese children and adolescents.

In order to assess whether there was publication bias, the visual inspection of the funnel plot revealed an absence of publication bias (see Figure 2). In addition, the regression coefficient of Egger’s test (*b* = −1.97, *SE* = 3.36, 95% CI = [−8.76, 4.81], *t*(41) = 0.59, *p* = 0.560) and the rank correlation coefficient of Begg’s test (*Z* = 0.36, *p* = 0.722) were non-significant, and the Fail-Safe N test showed that 26,233 additional studies of the associations between unsociability and peer problems would be required to change the significant overall effect size in the present meta-analysis, which was larger than the 5k + 10 limit [60]. Thus, there was no statistically significant publication bias in the selected studies.

### 4.2. Overall Effect Sizes

The overall random effects estimate showed that the correlations between unsociability and peer problems were *r* = 0.32, 95% CI = [0.248, 0.384], and *p* < 0.001. Thus, among Chinese children and adolescents, unsociability was positively and moderately associated with their peer problems.

### 4.3. Moderator Analysis

The results of the categorical moderator analysis are presented in Table 2. Results indicated a significant moderating effect of the living areas (urban vs. suburban vs. rural), *Q* (*df* = 2) = 30.13 and *p* < 0.001. Specifically, the associations between unsociability and peer problems were larger in suburban samples (*r* = 0.50) than in urban (*r* = 0.23) and rural (*r* = 0.46) samples. Also, informants of unsociability, *Q* (*df* = 2) = 123.77 and *p* < 0.001, and informants of peer problems, *Q* (*df* = 2) = 13.48 and *p* = 0.001, moderated the associations between unsociability and peer problems significantly. Specifically, the effect size of the associations was larger when unsociability was measured via peer nominations (*r* = 0.51) than via self-reports (*r* = 0.14) and parent ratings (*r* = 0.23). In addition, the effect size of the associations was larger when peer problems were measured via self-reports (*r* = 0.39) than via peer nominations (*r* = 0.34) and teacher ratings (*r* = 0.17). The study design and age group did not significantly moderate the associations between unsociability and peer problems in a Chinese sample. Moreover, meta-regression analysis showed that the percentage of boys was a trending significant moderator of the effect size, *b* = 0.004, *SE* = 0.002, 95% CI = [−0.0007, −0.0088], *Z* = 1.67, and *p* = 0.096.

## 5. Discussion

The present study conducted a meta-analysis to systematically examine the associations between unsociability and peer problems in Chinese children and adolescents. Overall, unsociability was found to be positively and moderately associated with peer problems in Chinese culture. In addition, there were significant moderating effects of living areas, informants of unsociability, and informants of peer problems among the associations, with stronger correlation coefficients in the subgroup of participants living in suburban areas, unsociability reported by peers, and peer problems reported by self.

### 5.1. Overall Associations between Unsociability and Peer Problems in Chinese Culture

The present meta-analysis found significant and positive associations between unsociability and peer problems in Chinese children and adolescents, which is consistent with previous empirical findings in Chinese culture [46,48,50,51,52,63]. Compared to the cross-cultural meta-analysis regarding the associations between social withdrawal and peer problems [53], the strength of the correlation coefficient was higher in the present study. This further reflects the motivational and cultural specificity of unsociability, especially in Chinese culture. As unsociability is conceptualized as the combination of low social approach motivation and low social avoidance motivation simultaneously [6], it represents the inherent preference and enjoyment of solitary activities. While unsociability may exert a positive influence on peer difficulties during adolescence [15], traditional Chinese culture that emphasizes interdependence and social affiliation may still exacerbate the maladaptive values caused by unsociability [40]. Thus, understanding the relation between unsociability and peer problems is of importance. 

Especially, the positive associations between unsociability and peer problems in the Chinese sample suggested that unsociable children may suffer adverse peer reactions and social difficulties in social interactions. This suggested that dropping out of social activities for a long time may give rise to social exclusion by peers [64]. From the perspective of cultural values, spending time alone violates the conventions of interdependence and social harmony and leads to more negative peer reactions [40]. The high sensitivity to interpersonal relationships within the group marks unsociable behaviors as anti-collective, selfish, and deviant [40]. Thus, in Chinese culture, unsociable children and adolescents are usually faced with more peer problems.

### 5.2. Moderators in the Associations between Unsociability and Peer Problems

To further explore the associations between unsociability and peer problems in Chinese culture, we conducted a series of moderation analyses to examine the moderating effect of study design, age group, gender, living areas, and informants of unsociability and peer problems. Inconsistent with our hypotheses, no significant moderating effects of age group and gender were found. This may be because, in collectivist cultures, social expectations and pressures may drive individuals of both sexes to seek harmony with the collective. Some researchers also speculated that peer pressure would be weakened from childhood to adolescence and solitude would be viewed as more positive and normative by adolescents [15,22,39]. However, in comparison to internalizing problems, peer problems seem to be a more direct negative outcome influenced by unsociability in Chinese culture. It has brought an extensive influence on the positive associations between unsociability and peer problems among Chinese children and adolescents, that is, both unsociable children and adolescents tend to have more peer problems. Consequently, it seems to be reasonable that there is no age difference among the relations between unsociability and peer problems. Moreover, the previous meta-analysis found the moderating effect of gender in the associations between social withdrawal and peer problems, which implied that social withdrawal was more harmful in Chinese boys than in girls [53]. Although no significant moderating effect of gender was found in the present meta-analysis, there is a trend toward stronger associations between unsociability and peer problems among Chinese boys. These results indicated a lower likelihood of tolerance for unsociable boys [14], as unsociability may be regarded as violating gender norms of male social assertion and dominance [47,50]. One possible explanation for such discrepancy could be that peer problems represent negative peer relationships. Even though, as we mentioned before, Chinese society highlights interdependence and group harmony and affiliation [10,13], it is equivalent for both genders. Correspondingly, boys and girls were educated neighborly and sociably connected with others, rather than in solitude by themselves [43]. Indeed, boys and girls are prone to interact with peers to obtain a higher level of peer preference, rather than being bullied or rejected by others. As a result, whether they are boys or girls, they all do not tend to interact with others in a negative way. Therefore, no difference was found among the relations between unsociability and peer problems.

A notable finding in the present meta-analysis was that the living areas moderated the associations between unsociability and peer problems in Chinese culture. Specifically, the correlation coefficient was stronger among those who live in suburban areas than in urban and rural areas. In the past several decades, there have been various socio-cultural changes influencing human behaviors and values, along with the globalization and urbanization process, which has an impact on Chinese culture as well [65]. As the population shifts from rural to urban areas, urban areas are exposed to more Western values (e.g., independence), where people have less maladjustment outcomes caused by unsociability, compared to those who living in rural areas [55]. While it was expected that children in rural areas would have stronger relations between unsociability and peer problems because of the emphasis on collectivism in rural areas (e.g., interpersonal harmony) [35], it was surprising that the associations in suburban areas were the strongest ones. When the financial and business centers in urban areas started to expand into suburban areas, people living in suburban areas were exposed to a mixture of traditional and modern cultural values [66]. The cultural and living gap between urban and suburban areas creates more challenges for unsociable children and adolescents to adapt to the new cultural values. Failure to fit may make children in suburban areas feel more cognitive conflicts and more vulnerable to maintain peer relationships in the process of urbanization [43,67]. As a result, the social structure and interpersonal relationships in suburban areas were strongly affected by globalization, and the effects of unsociability on peer problems turned out to be the highest ones.

In addition, we found that the informants of unsociability and peer problems significantly moderated the associations between unsociability and peer problems in Chinese children and adolescents. The correlation coefficient was higher when unsociability was measured via peer nominations and peer problems were measured via self-reports. Generally, unsociability was measured by the Revised Class Play [68,69], which supplies an “inside perspective” to report one’s engaging in peer interactions from the perspective of peers [70]. Compared to self-reports, teacher ratings [71], peer nominations [72], and parent ratings [73] may provide additional information of the children and adolescents and reduce some of the disadvantages of self-reports (e.g., socially desirable responding). However, ratings from others are also thought to be inappropriate for measuring personality information [23] and motivational tendencies [74].

This finding revealed that there is a higher likelihood of rejection or victimization when peers consider their classmates to be more unsociable, because they may be particularly regarded as failing to meet social expectations for group affiliation in the Chinese context. Self-reported peer problems were measured with different scales, such as the Pathways Project [75] and the multidimensional peer-victimization scale [76]. Self-reports have direct access to their subjective experience of being rejected, victimized, and excluded by peers and avoid bias from other possible informants. The results showed that children and adolescents who reported more experiences of peer problems had a higher level of unsociability, as the negative peer relationship may keep them from being involved in social interactions. Thus, as mentioned by Mundelsee and Jurkowski [77], using both adequate measures and appropriate statistical approaches to examine the links between shyness and sociability may provide more solid but surprising evidence, such as the negative relations between unsociability and peer problems in Western cultures.

### 5.3. Limitations and Future Directions

The present meta-analysis systematically examined the associations between unsociability and peer problems in Chinese children and adolescents and further explored the potential moderators among these associations. However, the findings should be interpreted with some limitations. First, the present study is unable to comprehend and elucidate the causal relationships between unsociability and peer problems. With increasing studies measuring unsociability and peer problems via longitudinal design, the future meta-analysis could gain a more thorough understanding of the longitudinal associations, especially for the effect of unsociability (the inherent preference for solitude) on later peer problems (acquired sociability). Second, although attention has been paid to the moderating effects of various informants of unsociability and peer problems, different measurements of these variables were not examined as the moderator in this meta-analysis, as the number of studies that used various measurements was relatively limited and unbalanced. Future studies could examine whether the measurements of unsociability and peer problems moderate the associations between them. Third, we found high heterogeneity of the effect sizes, suggesting that other potential factors need to be tested. Future meta-analysis could find more related empirical studies to enlarge the number of effect sizes and consider more moderators (e.g., children’s personality, socioeconomic status, and parenting style) to explore the psychological mechanisms of the associations between unsociability and peer problems. Meanwhile, the number of research studies on this topic is limited and more emphasis is needed to encourage more empirical studies in the scientific community. Finally, the majority of existing findings have utilized questionnaires to examine the associations and their potential psychological processes [46,50,51,52,63]. However, some studies have tried to examine social withdrawal with neural techniques. For example, Deng and colleagues discussed the links between social avoidance (i.e., another subtype of social withdrawal) and frontal alpha asymmetry when processing emotional facial stimuli, providing cognitive neuroscience evidence for socially withdrawn individuals [78]. Future meta-analyses could focus on studies of unsociability and peer problems with neuroimaging techniques (e.g., EEG, fMRI, and fNIRS) to identify the common and unique neural mechanisms across different studies.

## 6. Conclusions

In conclusion, the present meta-analysis summarizes the existing evidence from 21 publications examining the associations between unsociability and peer problems in Chinese children and adolescents. In general, unsociability was moderately and positively associated with peer problems. Hence, Chinese unsociable children and adolescents are particularly at risk of experiencing peer problems (e.g., peer rejection, peer victimization, and peer exclusion) during peer interactions. Moreover, the strength of the associations was moderated by living areas, informants of unsociability, and peer problems in Chinese culture. In particular, the associations between unsociability and peer problems were stronger among participants living in suburban areas, unsociability measured by peer nominations, and peer problems measured by self-reports. Findings indicate unique differences in the pattern of the associations between unsociability and problems and provide a more nuanced understanding of how living areas and informants influence adjustment outcomes under different conditions.

## Figures and Tables

**Table 1:** Characteristics of included studies.

First Author and Year	*N*	Percentage of Boys (%)	Living Areas	Age Group	Study Design	Informants of Unsociability	Informants of Peer Problems	Variable Labels of Unsociability	Variable Labels of Peer Problems	Effect Size (*r*)
Written in Chinese										
Ding, 2018	660	52.58	Urban	/	Cross-sectional	self-report	peer nomination	unsociability	peer problems	0.12
Gan, 2022	1125	49.07	/	Adolescent	Cross-sectional	self-report	self-report	unsociability	peer aggression	0.22
Yuan, 2014a	766	50.78	Suburban	Adolescent	Longitudinal	peer nomination	peer nomination	unsociability	peer victimization	0.62
Yuan, 2014b	766	50.78	Suburban	Adolescent	Longitudinal	peer nomination	peer nomination	unsociability	peer victimization	0.67
Yuan, 2014c	766	50.78	Suburban	Adolescent	Longitudinal	peer nomination	peer nomination	unsociability	peer victimization	0.55
Yuan, 2014d	766	50.78	Suburban	Adolescent	Longitudinal	peer nomination	peer nomination	unsociability	peer rejection	0.52
Yuan, 2014e	766	50.78	Suburban	Adolescent	Longitudinal	peer nomination	peer nomination	unsociability	peer rejection	0.54
Yuan, 2014f	766	50.78	Suburban	Adolescent	Longitudinal	peer nomination	peer nomination	unsociability	peer rejection	0.49
Zhu, 2018	221	/	Urban	Children	Cross-sectional	self-report	teacher rating	preference for solitude	peer rejection	0.20
Zhu, 2020	211	53.08	Urban	Children	Cross-sectional	parent rating	teacher rating	unsociability	peer rejection	0.21
Written in English										
Chen, 2011	820	53.9	Rural	Children	Cross-sectional	peer nomination	peer nomination	unsociability	peer rejection	0.45
Chen, 2015a	247	100	Urban	Children	Cross-sectional	peer nomination	peer nomination	unsociability	peer victimization	0.43
Chen, 2015b	247	100	Urban	Children	Cross-sectional	peer nomination	peer nomination	unsociability	peer rejection	0.68
Chen, 2015c	240	0	Urban	Children	Cross-sectional	peer nomination	peer nomination	unsociability	peer victimization	0.17
Chen, 2015d	240	0	Urban	Children	Cross-sectional	peer nomination	peer nomination	unsociability	peer rejection	0.24
Coplan, 2016	1344	51.94	Urban	Adolescent	Cross-sectional	self-report	peer nomination	preference for solitude	peer victimization	0.08
Ding, 2014	787	56.42	Urban	/	Cross-sectional	peer nomination	peer nomination	unsociability	peer problems	0.51
Ding, 2019a	601	53.41	Urban	/	Longitudinal	self-report	peer nomination	unsociability	peer rejection	0.04
Ding, 2019b	601	53.41	Urban	/	Longitudinal	self-report	peer nomination	unsociability	peer rejection	0.09
Ding, 2019c	601	53.41	Urban	/	Longitudinal	self-report	peer nomination	unsociability	peer victimization	0.10
Ding, 2019d	601	53.41	Urban	/	Longitudinal	self-report	peer nomination	unsociability	peer victimization	0.10
Ding, 2022a	578	53.63	Urban	/	Longitudinal	self-report	peer nomination	unsociability	peer rejection	0.03
Ding, 2022b	578	53.63	Urban	/	Longitudinal	self-report	peer nomination	unsociability	peer victimization	0.09
Ding, 2023a	571	54.82	Urban	Children	Cross-sectional	self-report	teacher rating	unsociability	peer problems	0.11
Ding, 2023b	345	53.04	Urban	Adolescent	Cross-sectional	self-report	teacher rating	unsociability	peer problems	0.15
Liu, 2014a	787	56.42	Urban	/	Longitudinal	peer nomination	peer nomination	unsociability	peer victimization	0.51
Liu, 2014b	787	56.42	Urban	/	Longitudinal	peer nomination	peer nomination	unsociability	peer victimization	0.59
Liu, 2017a	564	48.23	Suburban	Children	Cross-sectional	peer nomination	peer nomination	unsociability	peer victimization	0.50
Liu, 2017b	462	53.25	Suburban	Adolescent	Cross-sectional	peer nomination	peer nomination	unsociability	peer victimization	0.46
Sang, 2018a	663	52.79	Urban	/	Cross-sectional	self-report	peer nomination	unsociability	peer problems	0.12
Sang, 2018b	663	52.79	Urban	/	Cross-sectional	self-report	teacher rating	unsociability	peer problems	0.10
Xiao, 2021a	445	50.11	Urban	Adolescent	Longitudinal	self-report	peer nomination	unsociability	peer problems	0.28
Xiao, 2021b	445	50.11	Urban	Adolescent	Longitudinal	self-report	peer nomination	unsociability	peer problems	0.10
Xiao, 2021c	445	50.11	Urban	Adolescent	Longitudinal	self-report	peer nomination	unsociability	peer problems	0.17
Zhang, 2018a	93	53.00	Urban	Adolescent	Cross-sectional	self-report	self-report	unsociability	perceived peer exclusion	0.45
Zhang, 2018b	229	52.00	Rural	Adolescent	Cross-sectional	self-report	self-report	unsociability	perceived peer exclusion	0.49
Zhang, 2018a	229	52.00	/	Adolescent	Cross-sectional	self-report	peer nomination	unsociability	peer rejection	−0.01
Zhang, 2018b	229	52.00	/	Adolescent	Cross-sectional	self-report	peer nomination	unsociability	peer exclusion	0.09
Zhang, 2018c	229	52.00	/	Adolescent	Cross-sectional	peer nomination	peer nomination	unsociability	peer rejection	0.48
Zhang, 2018d	229	52.00	/	Adolescent	Cross-sectional	peer nomination	peer nomination	unsociability	peer exclusion	0.54
Zhu, 2020	487	58.11	Suburban	Children	Cross-sectional	parent rating	teacher rating	unsociability	peer exclusion	0.27
Zhu, 2021	194	52.58	Urban	Children	Cross-sectional	parent rating	teacher rating	unsociability	peer exclusion	0.12
Zhu, 2022	256	48.83	Suburban	Children	Cross-sectional	parent rating	teacher rating	unsociability	peer exclusion	0.26

**Table 2:** Results of the moderators for the associations between unsociability and peer problems in Chinese children and adolescents.

Moderator	Heterogeneity Analyses			Category	*K*	Effect Size				
	*Q*	*df*	*p*			*r*	95% CI		*Z*	*p*
Study design	0.453	1	0.501	Cross-sectional	26	0.297	0.222	0.369	7.449	0.000
				Longitudinal	17	0.346	0.221	0.459	5.215	0.000
Age group	0.731	1	0.392	Children	12	0.317	0.208	0.419	5.474	0.000
				Adolescents	19	0.381	0.276	0.477	6.664	0.000
Living areas	30.126	2	0.000	Urban	26	0.233	0.151	0.312	5.450	0.000
				Suburban	10	0.501	0.426	0.568	11.310	0.000
				Rural	2	0.459	0.410	0.505	16.013	0.000
Informants of unsociability	123.772	2	0.000	Self-reports	21	0.141	0.100	0.182	6.663	0.000
				Peer nominations	18	0.510	0.463	0.554	17.881	0.000
				Parent ratings	4	0.228	0.165	0.290	6.913	0.000
Informants of peer problems	13.479	2	0.001	Self-reports	3	0.386	0.171	0.565	3.413	0.001
				Peer nominations	32	0.344	0.259	0.423	7.561	0.000
				Teacher ratings	8	0.174	0.121	0.225	6.413	0.000

## Data Availability

The datasets used and analyzed in the study are available from the corresponding author upon reasonable request.
